# Life as an early career researcher: interview with Catherine Martel

**DOI:** 10.4155/fsoa-2016-0011

**Published:** 2016-02-12

**Authors:** Catherine Martel

**Affiliations:** 1 Faculty of Medicine, Université de Montréal, Montreal Heart Institute, 5000, Belanger Street, Montreal, Quebec, H1T 1C8, Canada

## Abstract

**Catherine Martel speaks to Francesca Lake, Managing Commissioning Editor:** Catherine Martel obtained her PhD from the Université de Montréal and pursued a postdoctoral fellowship first at Mount Sinai School of Medicine in New York (NY, USA), then at Washington University School of Medicine in St Louis (MO, USA), and obtained the Junior Investigator Award for Women from the Arteriosclerosis, Thrombosis and Vascular Biology council of the American Heart Association. Her postdoctoral work is certainly groundbreaking and brings forward new considerations in the field: she discovered that the lymphatic vessel route, the network that runs in parallel with the blood vessels, is critical for removing cholesterol from multiple tissues, including the aortic wall. In 2013, she joined the Arteriosclerosis, Thrombosis and Vascular Biology Early Career Committee, eager to bring a Canadian perspective to the group and get involved in council activities. Since 2014, she is an Assistant Professor at the Department of Medicine at the Université de Montréal, and a research scientist at the Montreal Heart Institute. Her research program now focuses on characterizing the physiopathologic role of the lymphatics in the initiation, progression and regression of atherosclerosis. Basic and translational research will allow her team to identify the causes of lymphatic dysfunction, and eventually target potential therapeutic strategies aiming at improving lymphatic function at the different levels of the atherothrombotic disease. You can follow her laboratory at @LaboMartel_ICM.

## Q Can you tell us about your career path to date?

A career in medical research is not easy. It takes a combination of elements such as ambition, hard work, ideas, integrity, patience and most of all, passion. If I have decided to become a research scientist, it is certainly because of the positive influence form my mentors, either laboratory directors or senior colleagues with whom I had the opportunity to evolve. I have learned so much from these people who, maybe even without noticing it, remarkably knew in their own way how to diffuse their passion for research to the new generation of scientists. I did my graduate studies at the Université de Montréal, in the laboratory of Dr Pierre Théroux (Montreal Heart Institute [MHI]). Mostly working in a translational research framework, my goal was to characterize the pathophysiological role of the complement system in patients presenting acute coronary syndromes. This first step in the world of science made me eager to learn more about the foundation of coronary artery diseases and led me to deepen my comprehension of the mechanisms underlying the onset and progression of atherosclerosis. I had therefore decided to pursue my next scientific career step in a more basic science-oriented (and ideally in an English-speaking, as my mother tongue is French) research environment. Thus, in 2009, I had the opportunity to start a postdoctoral training at the Immunology Institute of Mount Sinai School of Medicine in New York (NY, USA), in the laboratory of Dr Gwen Randolph, a former pillar in the laboratory of late Dr Ralph Steinman, recipient of the 2011 Nobel Prize in Medicine. In May 2011, Dr Randolph had decided to relocate to the Pathology and Immunology Department at the Washington University School of Medicine in St Louis (MO, USA), a significant challenge in my career that I would later value as an opportunity to acquire laboratory management skills. Science-wise, my postdoctoral training allowed me to extend my proficiency in cardiology and immunology by including novel ways to investigate cholesterol trafficking out of atherosclerotic plaques. This led to the discovery of a new prerequisite player in the modulation of cholesterol removal from the artery wall: the lymphatic system. On a more personal level, my working experience in the USA allowed me to meet with extraordinary international scientists, who are now close collaborators and friends. Inspired and encouraged by them, I had thus decided to pursue my path career as an independent scientist in academia.

## Q Can you tell us more about what your current position entails, & what you are working on?

In early 2014, I initiated my career as a Principal Investigator at the MHI and was appointed with the Faculty of Medicine at Université de Montréal. I was attracted by this research center as I sensed a great potential to therein develop an innovative translational research program. The MHI is an ultraspecialized hospital center dedicated to care, research, prevention, rehabilitation and the assessment of new technologies in cardiology. On the day of my interview, meeting with the other basic and clinical scientists convinced me that eagerness for meaningful and successful research was at the heart of everyone’s priority at the MHI. Thus, supported by my previous mentors who are eager to see me progress as an independent scientist, I am now pleased to bring my expertise to this multidisciplinary group in Canada, by providing new insights on the mechanism underlying atherosclerosis progression. Supported by strong preliminary results, my emerging team and I are proposing novel concepts placing at the forefront the lymphatic system in the progression of atherosclerosis. As a premise to our intensive and collaborative work, we have already secured collaborations with local and international scientists who are bringing a specific expertise in vessel and platelet physiology, lipoprotein metabolism and lipid profiling, just to name a few. Working in a multidisciplinary environment (basic and clinical sciences) allows the conciliation of our basic science research program to a clinical research endeavor to explore the correlation between lymphatic function and atherosclerosis in patients. Overall, we hope to bring forward novel perspectives that will lead to new therapeutic targets in the treatment of atherosclerosis and to impact favorably the health and quality of life of patients with cardiovascular heart disease.

## Q Can you tell us about your work with the Arteriosclerosis, Thrombosis and Vascular Biology’s Early Career Committee?

As a graduate or a postgraduate trainee, I was attending meetings, often wondering if young, early career scientists were involved in the leadership committees of international conferences. Quite soon in my career, I felt the need to speak up not only for myself but also for junior investigators who needed more guidance, new challenges or new career opportunities. I was a postdoctoral fellow when I first joined, in July 2013, the Early Career Committee of the the Arteriosclerosis, Thrombosis and Vascular Biology (ATVB) council of the American Heart Association (AHA) [1]. Becoming an active member of this committee was a breath of fresh air, allowing me to interact with scientists who were going through a similar career path, reaching for the same goal with the same drive: passion for science and health research. The ATVB Council has established an Early Career Committee to foster the development of trainees and early career professionals in the field of arteriosclerosis, thrombosis and vascular biology, including research scientists and clinicians. We are a small group (˜ten members) of scientists at the early stage of our career, coming from diverse backgrounds and working all together with the AHA/ATVB Leadership and staff to organize early career ATVB networking events, plan Early Career Symposium at AHA National Fall and ATVB spring meetings and set up activities that benefit early career scientists. As an early career individual myself, I really appreciate receiving advice form more senior scientists on various aspects of our work. Therefore, as I greatly value the partnership between mentors and mentees, I have joined, in 2015, the AHA/ATVB Council Mentoring Program. The goal of this program is to reach out to early career scientists who are members of the ATVB Council and provide networking opportunities by introducing early career researchers to leaders in all ATVB Council Member areas of interest.

## Q What do you find most rewarding about your work?

Starting a laboratory is certainly relentless: you need to be a good technician (because you often start off as being your own tech!), a convincing writer, an incredible thinker and… a great mentor. Being trained as a scientist involves that you accentuate your knowledge in science *per se*. However, it is quite rare that our training involves pedagogy classes. Even though I have learned to perceive public speeches as an exciting individual challenge, I had been wondering, before starting my own lab, if I would be able to transmit my knowledge to students, one-to-one. Albeit the special programs I took to improve my teaching and communication skills (both at Mount Sinai hospital in New York City and at Université de Montréal), I truly believe that success in knowledge translation resides in the desire and heart you put in your work. I believe this is contagious. Working in a stimulating and joyful environment is to me the most important aspect to consider when managing a laboratory. It is important that my students and techs are happy in what they are doing. So I would say that is the answer to your question: what is the most rewarding for me is to obtain good publishable results, produced in a trustful environment by students I can be proud of. The success of my students is definitely an aspect that I value a lot. My goal as a young evolving new investigator is to bring to the training environment not only scientific expertise but also passion for research.

## Q What would you say are the biggest challenges facing early-career researchers, from your point of view?

Getting funded. When I took the decision to pursue my path in academia in Canada, I knew it was not going to be easy, but I also knew that this career is what I wanted the most. The very first month I became an independent scientist, I turned into Shakespeare. I had to start writing and writing. Writing my own grants and applying for external funding and new investigator awards. In those applications, they ask you to show you productivity as an ‘independent’ Principal Investigator. This means you need to publish, ideally, as a senior author and without your former mentor as a coauthor. Not an easy task, considering that you have probably just left your postdoctoral training to transition to your faculty position, and that you are now ‘physically’ setting up your laboratory, trying to get innovative preliminary data (at one point, I thought about changing field and going into gene therapy to grow an extra pair of arms and an extra brain…). Then you apply, and apply, and apply to the funding agencies. You receive good scores, even qualified as ‘excellent’ or ‘outstanding', but you still might not get funded (there is a funding rate of 8–15% for some agencies)! And then, to cheer you up, you keep in mind that (apparently) Michael Jordan was cut from the basketball team as a high-school sophomore. Years later, he became the greatest basketball player of all time.

My team’s efforts started paying off last summer, when I received my first grant as an independent scientist: I was pleased to be one of the seven recipients to receive the Banting Research Foundation Discovery Award. This grant is “awarded to outstanding new investigators in all areas of medical research who are within the first 3 years of their independent appointment at a University in Canada” [2]. This grant is allowing us to deepen our knowledge in our specific field, and produce data that will be the premise for other grant applications.

## Q How would you suggest these are tackled?

Of course, having more funding available would certainly help. However, on a more realistic basis for young scientists like me, what I would suggest is to make sure that the postdoctoral trainees are well armed to face the heavy administrative tasks that they will have to face as Principal Investigators. Months, and even years before accessing their faculty positions, the candidates should be strategically coached and aware of what is coming ahead.

## Q If you could go back 5 years, what advice would you give to your younger-self in terms of your career?

I would tell myself what I tell my students: put your heart in everything you are doing. Believe in yourself, but allow yourself to ask questions, to admit that you do not know everything. Ask for help when you need it. Surround yourself with trustable, joyful and smart people. And, most of all, I would remind myself that quote of Victor Hugo: “Perseverance, secret of all triumphs.”
